# A Modified Technique for Transcatheter Pulmonary Valve Implantation of SAPIEN 3 Valves in Large Right Ventricular Outflow Tract: A Matched Comparison Study

**DOI:** 10.3390/jcm12247656

**Published:** 2023-12-13

**Authors:** Ali Houeijeh, Clément Karsenty, Nicolas Combes, Clément Batteux, Florence Lecerf, Frederic Remy, Estibaliz Valdeolmillos, Jérôme Petit, Sébastien Hascoet

**Affiliations:** 1 Hôpital Marie Lannelongue, Groupe Hospitalier Paris Saint Joseph, Centre de Référence Cardiopathies Congénitales Complexes M3C, Faculté de Médecine, Université Paris Saclay, BME Lab, 92350 Le Plessis-Robinson, France; karsenty.cl@chu-toulouse.fr (C.K.); n.combes@ghpsj.fr (N.C.); c.batteux@ghpsj.fr (C.B.); f.lecerf@ghpsj.fr (F.L.); f.remy@ghpsj.fr (F.R.); e.valdeolmillos@ghspj.fr (E.V.); j.petit@ghpsj.fr (J.P.); s.hascoet@ghpsj.fr (S.H.); 2 Pediatric Cardiology Unit, Lille University Hospital, Laboratoire EA4489, Lille II University, 59000 Lille, France; 3 Cardiologie Pédiatrique et Congénitale, Université de Toulouse, Hôpital des Enfants, CHU de Toulouse, 31300 Toulouse, France; 4 Clinique Pasteur, 31300 Toulouse, France; 5 Inserm UMRS999, Université Paris Saclay, 92350 Le Plessis-Robinson, France

**Keywords:** pulmonary valve, infective endocarditis, melody, SAPIEN, congenital heart disease

## Abstract

Introduction: Percutaneous pulmonary valve implantation (PPVI) with a SAPIEN 3 valve is effective for treating treat right ventricle outflow (RVOT) dysfunction. A modified technique was developed without prestenting using a protective valve delivery method. We aimed to compare the procedural results of the modified technique group (MTG) to those of patients in a conventional technique group (CTG). Methods: We designed a matched before–after study. All consecutive PPVI with SAPIEN 3 performed in the MTG over 9 months were matched, based on the RVOT type and size, to consecutive procedures performed previously with SAPIEN 3. Results: A total of 54 patients were included, equally distributed in the two groups. The sizes of the SAPIEN 3 valves were 23 mm (n = 9), 26 mm (n = 9), 29 mm (n = 36). The two groups were similar regarding demographic data, RVOT type, and pre-procedure hemodynamics. PPVI was performed in a single procedure in all patients of the MTG, whereas six (22.2%) patients of the CTG group underwent prestenting as a first step and valve implantation later (*p* = 0.02). The procedures were successful in all cases. Stent embolization was reported in two patients (7.4%) in the CTG, which were impacted in pulmonary arteries. In one case (3.7%), in the MTG, an unstable 29 mm SAPIEN 3 valve was stabilized with two stents and additional valve-in-valve implantation. The hemodynamics results were good in all cases, without significant differences between the two groups. The procedures’ durations and fluoroscopy times were significantly reduced in the MTG (48.1 versus 82.6 min, *p* < 0.0001; 15.2 versus 29.8 min, *p* = 0.0002). During follow-up, neither stent fracture nor valve dysfunction was noticed in either group. Conclusion: PPVI without prestenting and with a protective delivery method of the SAPIEN 3 valve significantly reduces the procedure’s complexity, the duration, and the irradiation while maintaining excellent hemodynamics results in selected cases.

## 1. Introduction

Percutaneous pulmonary valve implantation (PPVI) is effective in treating right ventricle outflow (RVOT) dysfunction. Jugular-vein-based valves mounted on a chromium platinum stent were used first in the conduit or native right ventricular outflow tract, ranging between 16 to 24 mm [1]. Prestenting was recommended in order to increase radial strength and the risk of valvular stent fracture during follow-up [2]. Thereafter, a bovine pericardium-based valve mounted on a chromium cobalt stent was used later in the pulmonary position, extending the feasibility of PPVI in large native RVOT up to 29 mm [3,4,5,6]. These valves and their delivery systems were originally developed for transaortic valve implantation, and cases of tricuspid valve injuries were reported while advancing the valve without covering along the right heart cavity [7]. Moreover, in large native RVOT, much is at stake is when obtaining a valve with good stability. There are fewer concerns regarding radial strength and risk of valve fracture, which has not yet been reported so far with this valve in the aortic position. Landing zone prestenting is challenging, and stent embolizations have been reported during the process of advancing the valve inside. Finally, there was a period of unavailability of large stents such as Andrastent XXL, Optimus XXL, or CP10Zig [8]. For all these reasons, prestenting became less used before the development of PPVI with these valves [9,10]. Since June 2019, we have modified our PPVI technique using a large-sheath GORE^®^ DrySeal Flex Introducer Sheath (W.L. Gore & Associates, Inc., Flagstaff, AZ, USA) [11] without systematic prestenting. We aim to report our preliminary experience and mid-term outcomes with this modified technique, with regard to safety and efficacy.

## 2. Methods

### 2.1. Study Design

We performed a prospective, single-center, matched before–after study. From the local database including all PPVI procedures, we selected consecutive patients who had undergone PPVI with a SAPIEN3 implanted using a modified technique (MTG) between June 2019 and February 2020. These patients were matched in a one-to-one setting to patients who had undergone SAPIEN 3 valve implantation with a conventional technique (CTG). Conventional PPVI cases were selected from July 2016 to December 2017. Matching was based on the RVOT type and valve diameter (Table S1, Supplementary Data). Patients requiring prestenting to treat RVOT complex stenosis were not included. The study protocol was approved by the local ethical committee (GERM IRB 00012157, reference 543, 27 October 2021). Informed consent was obtained from all patients.

### 2.2. Percutaneous Pulmonary Valve Implantation Protocol

PPVI was indicated based on international guidelines [12,13]. Symptomatic patients or those with evidence of RV or left ventricular systolic dysfunction; severe RV enlargement with an indexed RV end-diastolic volume of greater than 160 mL/m^2^; or an indexed RV end-systolic volume of greater than 80 mL/m^2^, along with decreases in exercise capacity, were eligible.

PPVI was performed using a SAPIEN 3 valve in all cases. The SAPIEN 3 valve is characterized by a trileaflet valve crafted from bovine pericardium. The valve is sutured to a cobalt–chromium stent frame with a cell design that minimizes the profile and improves the radial strength. Additionally, the SAPIEN 3 has an outer polyethylene terephthalate skirt which decreases the incidence of paravalvular leak. The SAPIEN 3 valve was introduced using the Commander delivery system. It obtained a CE mark in 2021. Good procedural and short-term outcomes following PPVI with the SAPIEN 3 valve have previously been reported [14,15,16].

**Figure 1:**
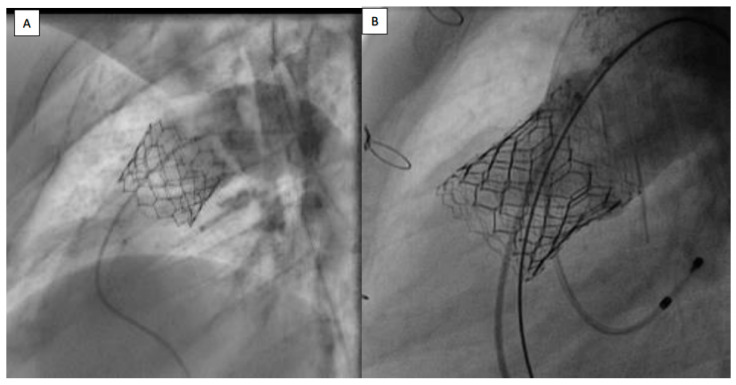
(**A**) Sapien 3 valve, implanted without prestenting in native patched RVOT. (**B**) Sapien 3 valve, implanted with prestenting in native patched RVOT. RVOT: right ventricle outflow tract.

The PPVI technique was modified by using a large, long 65 cm GORE^®^ DrySeal Flex Introducer Sheath (W.L. Gore & Associates, Inc., Flagstaff, AZ, USA) to safely advance the SAPIEN 3 valve into the landing zone along the right heart cavities [11]. Prestenting was avoided in case of regurgitant RVOT (conduit or native RVOT) without severe stenosis when a stable landing zone measuring less than 29 mm could be demonstrated during the preprocedural CT scan and during the compliant balloon testing [17]. Valves were delivered using a Commander^®^ delivery system in all cases (Figure 1).

The conventional technique used in the control cohort included SAPIEN 3 valve implantation delivered onto a prestented landing zone using the Commander^®^ delivery system, but without using the Dry Seal sheath. Patients who had PPVI with systematic prestenting and Dry Seal sheath were not included in this study, since they were reported in a previous study [14] explaining the gap between the two inclusion periods.

### 2.3. Statistical Analysis

The analysis involved comparing the two management techniques. The patients and clinical characteristics were compared using non-parametric statistics to address small numbers and distribution abnormalities. Propensity scores were estimated using logistic regression, with 1:1 nearest-neighbor matching. The propensity score model included the RVOT type and size.

## 3. Results

### 3.1. Patients’ Selection

We included 27 patients who underwent SAPIEN 3 valve implantation using the modified technique matched to 27 patients who underwent conventional PPVI. PPVI was performed in 35 patients during the period of inclusion of the MTG, eight of whom patients were excluded. Five patients had pre-stenting for severe stenotic conduits; one patient had associated left pulmonary artery stenting; and two patients had PPVI without the Dry Seal sheath, which was unavailable during the session.

During the inclusion period of the CTG, 31 patients underwent PPVI, of whom 4 patients were excluded. Two patients had severe conduit stenosis, one patient had associated intervention (patent foramen ovale closure), and one patient had not have matched case in the MTG. This patient had an uneventful and successful procedure outcome.

### 3.2. Baseline Characteristics

Baseline demographics and procedural data are reported in Table 1. The two groups were similar regarding the demographic data, RVOT type, and pre-procedure hemodynamic conditions. Severe pulmonary regurgitation was the most frequent lesion (16 cases in the CTG group and 19 cases in the MTG group). Hemodynamic moderate stenosis (pic gradient > 20 mmHg) was observed in 11 cases in the CTG and 8 cases in the MTG group. In all of them, the stenosis length was short. Hemodynamic and pressure data were similar within the two groups.

### 3.3. Procedures

The PPVI procedures were performed under general anesthesia in all cases in the CTG and in 25 (92.6%) cases in the MTG group (*p* = 0.49). Two procedures were performed under local anesthesia given a critical pre-operative status.

PPVI was performed in a single procedure in all patients of the MTG group, whereas PPVI was performed in two steps in six patients of the CTG group. In these cases, prestenting was carried out in a first procedure and PPVI in a second (*p* = 0.02).

### 3.4. Procedure Efficacy

The post-procedure hemodynamic data were similar between the two groups. Despite, the duration of fluoroscopy and the dose of the first catheterization were not considered in the CTG, and significant reductions in the procedure time and irradiation doses were observed in the MTG (Table 2).

### 3.5. Procedure Safety

Stent embolization was reported in two patients. It was impacted in the right pulmonary artery in one case and in the left pulmonary artery in the second case, without further consequence. In these two cases, another stent was then positioned in the landing zone using a larger balloon, and PPVI was successfully performed in a second procedure few months later.

In the MTG, a 29 mm SAPIEN 3 valve was unstable in a large, native RVOT and was stabilized with two stents. A 29 mm SAPIEN 3 valve was finally implanted with good results. Post-PPVI, acute pulmonary edema was observed in one patient because of severe left ventricular dysfunction, which was present before PPVI. The pulmonary edema was resolved quickly under diuretics therapy.

No tricuspid valve injury was reported in either of the two groups.

### 3.6. Procedure Cost

The hospitalization stay durations were similar in both groups; 3 days (3–3 days) in the MTG group and 3 days (3–3 days) in the CTG group. One patient had a longer stay in the MTG group because of severe left ventricle dysfunction, which was present before the intervention. The standard cost for the material used for the procedure used for the CTG is EUR 16,449, and for the MTG, EUR 15,101.

### 3.7. Follow-Up

Post-procedural data were similar between the two groups. The median post-procedural transpulmonary valve maximal velocity was 1.9 m/s [1.7–2.4], without significant differences among the two groups (2.0 in CTG versus 1.8 in MTG, *p* = 0.1). In the CTG, trivial pulmonary regurgitation was reported in six cases (22.2%) and mild in one patient (3.7%). In the MTG, trivial pulmonary regurgitation was not reported, and mild pulmonary regurgitation was reported in one patient (3.7%). Pulmonary regurgitation was most often reported in the CTG (*p* = 0.023).

After a median follow-up duration of 40.1 [25.8–40.9] months for the CTG and 12.9 [7.9–19.3] for the MTG group, no cases of valve dysfunction, valve stent fracture, secondary valve replacement, infective endocarditis, or death were reported in either of the two groups. The median transpulmonary valve maximal velocity at the last follow-up was 2.2 m/s [1.8–2.6], without a significant difference between the two groups (2.2 versus 2.2, *p* = 1.0). Trivial pulmonary regurgitation was reported in eight cases (14.8%) and mild in one patient (1.9%), without significant difference between the two groups (*p* = 0.142). The patients were either in NYHA 1 (81.5%) or 2 (18.5%).

## 4. Discussion

In this single-center, before–after, matched cohort study, we illustrate the feasibility and efficacy of PPVI, with SAPIEN 3 delivered using a long protective sheath in large native non-stented RVOT. This study illustrates that these two modifications to the conventional PPVI protocol considerably reduced the procedure time in these selected cases, as illustrated by a decrease in fluoroscopy irradiation with efficient, immediate midterm results.

Prestenting has been systematically performed before PPVI to increase the radial strength of the landing zone after reports of occurrences of Melody valve stent fractures during follow-up [18,19]. So far, no SAPIEN 3 stent fractures have been reported. Similarly, no significant paravalvular leaks nor stent fractures were registered in this study, in contrast with the studies on the Melody valve where stent fractures were reported in up to 20% of cases, mainly in non-prestented RVOT [2]. Previous reports on Sapien valve implantation have confirmed the absence of stent fracture at midterm follow-up, whether or not presenting was performed [10,14,16,20,21]. This modified technique has also been adopted in other centers [9,10] as illustrated in a large multicenter study including 774 patients, in which nearly half of patients with Sapien 3 valve implantation did not have prestenting with no complications [16]. The absence of stent fractures reported with the Sapien valve may be related to its mechanical properties, as well as to the RVOT characteristics compatible with these valves. The cobalt–chrome stent design has a high radial strength and robustness compared to the platinum–iridium stent of the Melody valve. In addition, the stent frame of the Sapien valve is shorter than that of the Melody valve. Consequently, it is less usual to for the valve to have an asymmetric shape after deployment, as this has been reported as a risk factor for stent dysfunction [22,23]. Finally, Sapien valves are expanded at a larger diameter than Melody valves, and this may also contribute to differences in radial strength. However, differences in RVOT characteristics in which the two types of valves are implanted certainly contributes to this result. Melody valves have mainly been implanted in small, stenotic, and often calcified conduits, exerting high radial strain on the stent. Currently, Sapien valves are often implanted in native large regurgitant RVOT with less external strain. Furthermore, when Sapien valves are implanted in small or stenotic conduits, stenting remains routinely performed to ensure the hemodynamic efficacy and safety of the procedure prior to valve implantation, as illustrated by the patients excluded from this study and from other cohorts [24].

In patients with large native RVOT, prestenting was often used to prepare a “landing zone” for valve implantation. Indeed, the main challenge in these patients is to obtain valve stability after its deployment. Moreover, the Sapien valve was developed to treat calcified aortic stenotic valves, whereas, large patched RVOT is characterized by an unpredictably wide range of extensibility and distensibility [17,25]. The distensibility of the RVOT is investigated using balloon testing prior to valve implantation. Since the maximal valve diameter of the Sapien 3 is 29 mm (this can be expanded by one more millimeter in diameter) a stable area within the RVOT below this threshold is expected. In this situation, prestenting creates a stable conduit for subsequent valve implantation. Prestenting is even sometimes performed using multiple stents to anchor the landing zone in a pulmonary artery (“Jailing technique”) or to decrease its maximal diameter (“Russian dolls technique”) [26]. However, when a solid area is identified within the RVOT, direct valve implantation without prestenting can alternatively be considered. Issues with these two strategies are illustrated in this study. On one hand, prestenting increases the complexity of the procedure, its duration, its cost, and the fluoroscopy duration. Entering the stent with the sheath and the valve can be challenging, with a risk of stent crushing and stent embolization [8]. To avoid this latter complication, which occurred in two cases in this study, PPVI was often performed in two steps. Prestenting is first performed and the valve is implanted a few months afterward, allowing the stent to be more adherent to the RVOT wall, but further complexifying the whole process. Valve implantation without prestenting considerably hastened the procedure’s duration and process. However, in one case, the implanted valve was unstable, needing to be impacted with multiple stents, and a second valve had to be inserted. The management of bare stent embolization is easier and more cost-effectiveness than the management of valve embolization. During a short period of time, we were forced to move from one technique to another given the shortage of long and large stents. Currently, CP10Z stents, Optimus XXL, and Andrastent XXL are large stents that can be efficiently used for prestenting. We have, thus, further evolved in our strategy. We consider direct valve implantation in regurgitant non-stenotic conduits in bioprosthesis (valve-in-valve) and in native RVOT when a non-distensible target landing zone is evidenced. Otherwise, prestenting is performed.

Self-expandable valves are being developed and increasingly used. They further expand the feasibility of PPVI for very large RVOT. This valve design does not require prestenting [27,28,29,30,31,32,33,34,35,36]. Indeed, the valve is embedded within a self-expandable nitinol stent frame with a memory-of-shape property. Another option that is being developed is a self-expandable nitinol platform (Alterra, Edwards lifesciences) implanted in the RVOT up to 35 mm, which would allow for secondary PPVI with the usual SAPIEN 3 valves [37].

The other significant modification was the use of a long, large sheath to advance the SAPIEN valve to the landing zone [11,38]. The delivery systems for the Sapien valves were adapted for the aortic position. The valve was uncovered to minimize the size of the sheath to be introduced into the artery. In the pulmonary position, venous access allows for the use of larger sheaths. Moreover, uncovered valve-induced lesions in the tricuspid valve were found in about 5% of cases [39]. Finally, the presence of the sheath beyond the landing zone could prevent the rare case of failure to advance the valve to the landing zone [40].

This study has some limitations; the first is the limited number of the patients in each group due to the monocentric design and relatively the short_term follow-up. The main limitation is the gap between the two periods of inclusion, which induces timing bias as a result of the evolution of the techniques and the experiences of the operators.

## 5. Conclusions

A modified technique of percutaneous implantation of a SAPIEN 3 valve in the pulmonary position, without prestenting and using a long delivery sheath, was safe and effective in selected eligible patients with maximal landing zone diameter < 29 mm and without significant extensibility. The modified technique reduces the procedure’s duration, thus decreasing irradiation and improving its cost-effectiveness.

## Figures and Tables

**Table 1:** Characteristics of the patients; hemodynamic and echocardiographic data of the two groups.

	Conventional Technique n = 27	Modified Technique n = 27	*p*-Value
Age (years), median [Q1;Q3]	30.0 [24.4–47.0]	31.0 [22.0–36.5]	0.42
Weight, mediane [Q1;Q3]	58.0 [52.5–73.2]	60.3 [51.3–77.4]	0.85
Height, mediane [Q1;Q3]	167 [162–173]	170 [159–177]	0.85
Male	14 (51.9%)	15 (55.6%)	0.78
Type of congenital heart diseases			1.00
Fallot	23 (85.2%)	22 (81.5%)	
Pulmonary valvar stenosis	1 (3.7%)	2 (7.4%)	
Pulmonary atresia with intact ventricular	1 (3.7%)	1 (3.7%)	
Troncus Arteriosus	1 (3.7%)	0 (0.0%)	
Transposition of great arteries	1 (3.7%)	0 (0.0%)	
Aortic valve stenosis + Ross procedure	0 (0.0%)	1 (3.7%)	
Carcinoid Pulmonary valve lesion	0 (0.0%)	1 (3.7%)	
Right ventricle outflow tract			0.97
Native	18 (66.7%)	18 (66.7%)	
Homograft	1 (3.7%)	2 (7.4%)	
Bioprosthetic valve	5 (18.5%)	4 (14.8%)	
Conduit	2 (7.4%)	2 (7.4%)	
Valve-in-valve	1 (3.7%)	1 (3.7%)	
NYHA status			0.85
1	8 (29.6%)	6 (22.2%)	
2	16 (59.3%)	16 (59.3%)	
3	3 (11.1%)	4 (14.8%)	
4	0 (0.0%)	1 (3.7%)	
Indications			0.09
Pulmonary regurgitation	16 (59.3%)	19 (70.4%)	
Pulmonary stenosis	1 (3.7%)	3 (11.1%)	
Mixed lesion	10 (37.0%)	5 (18.5%)	

**Table 2:** Procedure characteristics in both groups, and follow-up data.

	Traditionnal Techniques n = 27	Modified Techniques n = 27	*p*-Value
Pre-procedure hemodynamic data			
Systolic aortic pressure	91 [83–105]	93 [83–100]	0.81
Systolic RV pressure	42 [34–52]	33 [30–48]	0.12
RV–pulmonary artery gradient	23 [13–34]	21 [10–32]	0.42
Procedure charecteristics			
Procedure time (min)	82.6 ± 32.8	48.1 ± 19.7	0.001
Fluoroscopy time (min)	29.8 ± 22.1	15.2 ± 7.9	<0.0001
Kerma	815.0 ± 755.9	341.5 ± 300.6	0.003
PDS	9543.3 ± 9726.9	3923.4 ± 3714.2	0.006
Post-procedure hemodynamic data			
Systolic RV pressure	34 [30–41]	32 [28–39]	0.89
RV–pulmonary artery gradient	7 [3–15]	6 [3–9]	0.78
Last fellow-up data			
RVOT Doppler peak velocity	2.2 [1.8–2.6]	2 [1.8–2.2]	0.68
TV Doppler peak gradient	40 [30–45]	35 [30–45]	0.81

RV: right ventricle, RVOT: right ventricle outflow tract, TV: tricuspid valve. Results are expressed as mean ± SD or median [1st, 3rd quartile].

## Data Availability

All data could be delivered by the corresponding authors after institutional approval within the respect of medical ethics.

## Supplementary Materials

The following supporting information can be downloaded at: https://www.mdpi.com/article/10.3390/jcm12247656/s1, Table S1: Features of the 27 matched patients. * minimal diameter measured during the procedure.
