# Supplementary material for: A Modified Technique for Transcatheter Pulmonary Valve Implantation of SAPIEN 3 Valves in Large Right Ventricular Outflow Tract: A Matched Comparison Study
Source: J Clin Med. 2023 Dec 13;12(24):7656. doi: 10.3390/jcm12247656 (PMC10743789; doi:10.3390/jcm12247656)
Supplement: Supplementary file 1 [file jcm-12-07656-s001.zip › jcm-2699041-supplementary.pdf]

Supplementary Materials

Table S1. features of the 27 matched patients.

	Conventional Technique Group			Modified Technique Group		
	RVOT type	Diameter*	SAPIEN 3 size	RVOT type	Diameter*	SAPIEN 3 size
1	Native	22	29	Native	26	29
2	Native	20,3	26	Native	30	29
3	Homograft	20	26	Homograft	21	29
4	Native	24	29	Native	24	29
5	Native	25	29	Native	29	29
6	Trifecta	23	26	Trifecta	23	26
7	Native	24	29	Native	25	29
8	Native	16,8	23	Native	18	23
9	Contegra	18	26	Homograft	20	26
10	Native	26	29	Native	26	29
11	Native	20,5	26	Native	21	26
12	Hancock	22	23	Hancock	22	23
13	Trifecta	21	23	Carpentier	21	23
14	Native	26	29	Native	26	29
15	Native	25	29	Native	29	29
16	Native	24	29	Native	26	29
17	Native	25	29	Native	26	29
18	Carpentier	23	29	Trifecta	27	29
19	Carpentier	21	23	Hancock	22	23
20	Melody	18	29	SAPIEN XT	23	23
21	Perimount	23	26	Trifecta	25	26
22	Native	25	29	Native	27	29
23	Native	26	29	Native	27	29
24	Native	24,5	29	Native	28	29
25	Native	25	29	Native	29	29
26	Native	22	29	Native	23	29
27	Native	22,6	29	Native	28	29

* minimal diameter measured during the procedure
